# Characterization of Key Aroma Compounds of *Zhuyeqing* by Aroma Extract Dilution Analysis, Quantitative Measurements, Aroma Recombination, and Omission Studies

**DOI:** 10.3390/foods14030344

**Published:** 2025-01-21

**Authors:** Lihua Wang, Ying Han, Xing Zhang, Xiaojuan Gao, Yan Xu, Qun Wu, Ke Tang

**Affiliations:** 1 Laboratory of Brewing Microbiology and Applied Enzymology, State Key Laboratory of Food Science & Technology, Key Laboratory of Industrial Biotechnology of Ministry of Education, School of Biotechnology, Jiangnan University, 1800 Lihu Ave, Wuxi 214122, China; wanglh0419@126.com (L.W.); yxu@jiangnan.edu.cn (Y.X.); 2 Laboratory of Analytical, Quality Inspection Center, Key Laboratory of Plant Extraction and Health of Chinese Lujiu (Shanxi), Shanxi Xinghuacun Fenjiu Distillery Co., Ltd., Fenyang 032205, Chinafjpjbyh@163.com (X.G.)

**Keywords:** *Zhuyeqing*, key aroma components, aroma extract dilution analysis, odor activity value, recombination, omission

## Abstract

*Zhuyeqing* is a flavored liquor with a unique flavor blended with *Qingxiangxing* Baijiu (Fenjiu) and botanical extracts. The aroma characteristics of *Zhuyeqing* were investigated using a sensomics approach. Ninety-three odorants, among them 64 odorants with flavor dilution (FD) ≥ 32, were confirmed in *Zhuyeqing* by gas chromatography-mass spectrometry/olfactometry (GC-MS/O) analysis. Quantitative analysis revealed that 22 odorants with odor activity values (OAVs) ≥ 1. Aroma recombination tests showed that 22 odorants with OAV ≥ 1 can recombine the aroma characteristics of *Zhuyeqing*; omission tests revealed that ethyl cinnamate, ethyl octanoate, ethyl acetate, β-damascenone, and eugenol with OAV ≥ 10 had significant effects on *Zhuyeqing*.

## 1. Introduction

**Figure 1:**
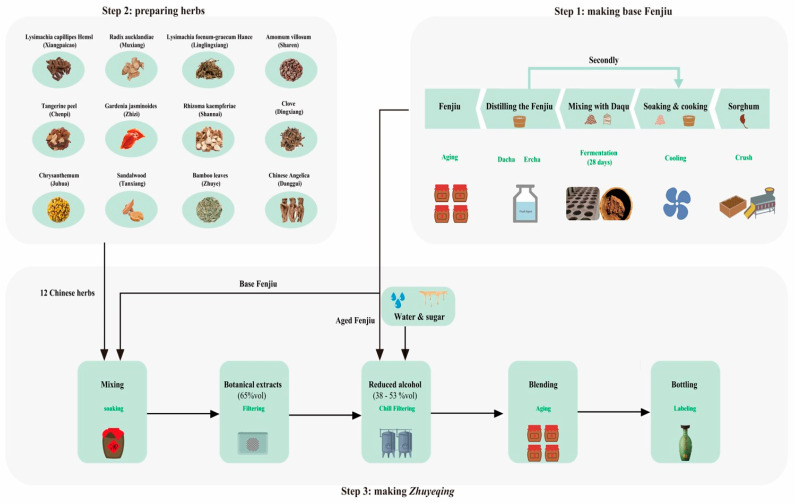
Process diagram for the production of *Zhuyeqing*.

*Zhuyeqing* is the most renowned Chinese functional and flavored distilled spirit with a distinctive flavor. This flavored liquor consists of botanical extracts with 12 Chinese herbs in the base liquor of Fenjiu [1]. Fenjiu is one of the most representative brands of *Qingxiangxing* Baijiu. The addition of these non-Fenjiu materials may introduce additional aromatic compounds to the Fenjiu matrix, significantly contributing to the flavor and mouthfeel of *Zhuyeqing*. Consequently, this unique production process imparts a distinctive aroma profile to *Zhuyeqing* (Figure 1), and the recipe is secret in China.

The aroma of alcoholic beverages is a critical factor influencing product quality and consumer preference [2,3]. Flavored alcoholic beverages are primarily categorized into two types: those based on fermented spirits (called flavored fermented spirits, FFS) and those based on distilled spirits (called flavored distilled spirits, FDS). Many aroma studies on flavored alcoholic beverages have mainly focused on flavored wine, a type of FFS. Vermouth, Bermet, Retsina, and Vesper are typically traditionally flavored wines [4]. Studies on the aroma of flavored wines have demonstrated that the aroma characteristics of flavored wines vary significantly depending on flavor additives [4]. Flavor additives can be categorized as herb-based [5,6,7], fruit-based (including grape-derived extracts like grape marc [8] and grape skin [9]), non-grape fruits [7,10,11,12], or other seldom-used materials such as rice [13]. Research has indicated that both the order of herb-based extract additions (pre- and post-fermentation) and the amount of extract additions can influence the particular aroma compounds and sensory properties of the resultant wine [5]. The floral and fruity aromas of wine can be masked by other scents introduced from herb-based extract additions, including medicinal, moldy/earthy, nutty, vegetal, solvent, and spice notes [6]. In contrast, the addition of fruit-based extracts significantly enhanced the overall fruity and floral aromas due to the increased concentrations of terpenes and esters [8,9,11,12], and the flavoring effect was also affected by the amount of extract added.

However, published studies on the aromatic characteristics of FDS are quite limited, focusing primarily on gins, *Zhizhonghe Wujiapi*, and Chinese *JingJiu*. Vichi, et al. [14] studied the volatile compositions of different distilled dry gins with geographical indications and the aroma profiles and key odorants in two gins with different botanicals from a German distillery. This research used headspace solid-phase microextraction (HS-SPME) coupled with gas chromatography-mass spectrometry (GC-MS). Buck, et al. [15] employed the *sensomics approach* to investigate the aroma profiles and key odorants in two gins with different botanicals sourced from a distillery in Germany. Similarly, Ma, et al. [16] and Sun, et al. [17] studied the aromatic characteristics of *Wujiapi* and Chinese *JingJiu*, respectively.

The sensomics approach, also known as molecular sensory science, involves discovering and accurately quantifying odor-active compounds, followed by aroma reconstitution and omission experiments [18]. This method has proven effective in identifying the aroma compounds responsible for the unique aroma profiles of various foods, such as Baijiu [19], tea [20], cheese [21], and broth [22]. *Zhuyeqing* is the most historically significant representative of *FDS.* The production process and selected herbs contribute to the unique aroma and flavor of *Zhuyeqing*. However, limited research exists on the aromatic characteristics of *Zhuyeqing*.

Consequently, the sensomics approach was used to study the aroma characteristics of *Zhuyeqing* in this paper and the purpose was as follows: (1) clarify the aroma composition of *Zhuyeqing* using aroma extract dilution assays (AEDA) connected with gas chromatography-mass spectrometry/olfactometry (GC-MS/O); (2) quantify the odor-active compounds by several quantitative approaches and calculate odor activity values (OAVs); and (3) construct a recombination and omission model of aroma to validate the key odorants that contribute to the odor of *Zhuyeqing*.

## 2. Materials and Methods

### 2.1. The Samples

The samples were produced based on traditional processes (as shown in Figure 1) and were supplied by Xinghuacun Fenjiu Distillery Co., Ltd. (Fenyang, China). The 12 Chinese herbals were mixed according to the secret recipe (step 2), soaked in base Fen produced via solid-state brewing technology (step 1), and filtered to obtain a 65% botanical extract after 21 days (step 3). Subsequently, the plant extracts were added to aged Fen wine in specific proportions, followed by the processes of reducing alcohol, cooling, filtration, and blending. The final *Zhuyeqing* product was obtained after aging, bottling, and labeling.

The representative samples were confirmed by sensory evaluation using a sensory panel composed of 10 nationally certified Chinese liquor tasters from the Laboratory of Xinghuacun Fenjiu Distillery Co., Ltd. A typical *Zhuyeqing* and a base Fenjiu (500 mL in each bottle, all with alcohol content of 45% *v*/*v*) were selected to conduct the sensory and gas chromatography- olfactometry (GC-O) analyses. The samples (three bottles per sample) were stored at 25 °C in the dark before use.

### 2.2. Reagents and Chemicals

Dichloromethane (HPLC grade, ≥99.8%) and absolute ethanol (HPLC grade, ≥99.8%) were purchased from Dikma Technologies Inc. (Beijing, China). Dichloromethane was redistilled prior to use. Sodium chloride (NaCl) and anhydrous sodium sulfate (Na_2_SO_4_) were obtained from China National Pharmaceutical Group Co. (Shanghai, China). Ultrapure water was obtained using a Milli-Q purification system (Millipore, Bedford, MA, USA).

All analytical standards and internal standards (ISs) (as shown in Table S1) with at least 95% purity were obtained from Aladdin Biochemical Technology Co., Ltd. (Beijing, China), Sigma-Aldrich Co., Ltd. (Shanghai, China), Innochem Science & Technology Co., Ltd. (Beijing, China), Macklin Biochemical Technology Co., Ltd. (Shanghai, China), Toronto Research Chemicals Inc. (Toronto, ON, Canada), TMRM Quality Inspection Technology Co., Ltd. (Changzhou, China), and ZZBIO Co., Ltd. (Shanghai, China). A C7-C30 n-alkane mixture (Dikma Technologies Inc., Beijing, China) was used to determine the retention indices (RIs).

### 2.3. Sensory Analysis

A sensory assessment panel (10 members with 3 males and 7 females, 27–40 years old) from Xinghuacun Fenjiu Distillery Co., Ltd. (Fenyang, China) was employed to obtain the aroma profile. Le nez du vin (Jean Lenoir, France) with 54 standard odor descriptors and extracted solutions with 12 traditional Chinese herbs of *Zhuyeqing* were used to train the evaluation panel. The particular training sessions were performed according to previously published procedures [23] as follows:

At first, participants provided sensory descriptors autonomously. Later, the panelists engaged in a discussion to determine the full list of descriptors from which the most cited descriptors were selected. Ultimately, 11 descriptors of aroma were selected for evaluation. The assessors rated the perceived strength of each descriptor in the sample on a scale of 0 (very weak) to 6 (very strong). Table 1 lists the reference solutions containing the chemicals and extractions.

After training, the panelists judged the samples (base Fenjiu and *Zhuyeqing*) at 25 °C. The tested samples (~20 mL) were filled in glasses and labeled with three-digit codes.

### 2.4. Comparative Aroma Extract Dilution Analysis of Zhuyeqing and Base Fenjiu

#### 2.4.1. Aroma Compound Extraction Methods

The liquid-liquid extraction (LLE) method was performed according to a published report [24], with minor modifications, to detect aroma compounds in *Zhuyeqing* and base Fenjiu. Each sample (50 mL) was diluted with 10% (*v*/*v*) ethanol and saturated with NaCl. Extraction was then carried out with 50 mL dichloromethane (CH_2_Cl_2_, 50 mL each time) for 5 min (repeated 3 times), and the collected organic phases were combined. Finally, anhydrous Na_2_SO_4_ was used to dry the organic phases overnight, and the sample was concentrated to 500 μL by nitrogen with a slow-flow stream and stored at −20 °C for use.

#### 2.4.2. Gas Chromatography-Mass Spectrometry/Olfactometry (GC-MS/O) Analysis

In this study, a gas chromatograph (Agilent 7890A) connected to a mass-selective detector (Agilent 5977B) and an olfactometry system (ODP 2, Gerstel, Mülheim an der Ruhr, Germany) (GC-MS/O) were used. A DB-FFAP (60 m × 0.25 mm i.d., 0.25 μm, Agilent, Santa Clara, CA, USA) and a DB-5 (30 m × 0.25 mm i.d., 0.25 μm, Agilent, Santa Clara, CA, USA) columns were used for compounds separation and perform the GC-O analysis. In splitless mode, 1 μL of the sample was injected at 250 °C with a 5 min solvent delay. The oven temperature settings were as follows: start at 45 °C and hold for 2 min, with a 4 °C/min ratio to increase to 80 °C, keep for 1 min, then with a 5 °C/min ratio to increase to 150 °C, keep 2 min, finally, with a 10 °C/min ratio to increase to 230 °C, hold for 10 min at 230 °C (DB-FFAP) or start at 45 °C and keep for 2 min, with a 5 °C/min ratio to increase to 150 °C, keep for 3 min, then with a 10 °C/min ratio to increase to 320 °C, and keep for 10 min at 320 °C (DB-5). The carrier gas for the column was helium (purity > 99.999%) at a flow rate of 1.5 mL/min. The quadrupole ionization energy was set at 70 eV in electron ionization mode, the temperature of the ion source was 250 °C, and the range of mass scan was from *m*/*z* 40 to 350. The sniffer port temperature was set at 250 °C for the entire duration of the GC-O. GC-O analysis was performed as previously reported [2]. Three experienced students (two females and one male) formed a team from our laboratory at Jiangnan University to carry out GC-O analysis. First, the evaluators analyzed the extracts on the DB-FFAP and DB-5 columns and noted each compound’s retention time and odor descriptors. Then, they discussed the odor descriptors of the compounds, verified them with chemical standards, and memorized these odor characteristics. Finally, AEDA was used to identify the significance of the odorants.

#### 2.4.3. Aroma Extract Dilution Analysis

Initially, AEDA was used to evaluate each aroma contribution detected by GC-MS/O and then to analyze the difference in aroma compounds between the *Zhuyeqing* and base Fenjiu samples.

The concentrated samples of *Zhuyeqing* and base Fenjiu were diluted in a 1:2 ratio with CH_2_Cl_2_ for AEDA. The flavor dilution (FD) value was defined as the highest dilution at which aroma compounds could be identified. Three experienced assessors (two females and one male) conducted the AEDA experiment. Each assessor repeated the analysis at least twice for each sample (including the original aroma extract and stepwise-diluted sample). The aroma components (Table 2) were identified by comparing the odor descriptors, mass spectrometry (MS), and retention indices (RIs) with those of real standards (Std). Referring to the modified Kovats method [25], the RIs of the odorants on the DB-FFAP and DB-5 columns were calculated from the retention times of the *n*-alkane (C7-C30) standards.

### 2.5. Quantitative Analysis of Aroma Compounds

Three instrumental analytical techniques with various extraction methods were used to establish targeted and accurate quantitative procedures based on the concentration ranges and the properties of the compounds. The standard curves of the horizontal coordinates were the concentration ratios of the target compound to the internal standard, and the vertical coordinate was the peak area ratio (Table 3). All calibration curves were within the linear range (R^2^ ≥ 0.99), and each sample was quantified in triplicate for accuracy.

#### 2.5.1. Liquid-Liquid Microextraction Combined with Gas Chromatography-Mass Spectrometry (LLME-GC-MS)

Thirty-one compounds with strong polarity and high concentrations were quantified by LLME-GC-MS, as previously reported [23]. Each sample (8 mL) was diluted with 10% ethanol (*v*/*v*), saturated with NaCl, and 50 μL of mixed ISs was added. The mixed ISs containing 1-butanol-d10 (IS1, 500.00 mg/L), ethyl octanoate-d15 (IS2, 500.00 mg/L), (±)-linalool-d3 (IS3, 100.00 mg/L), l-menthol (IS4, 100.00 mg/L), 2-ethylbutyric acid (IS5, 300.00 mg/L), 2-methoxyphenol-d3 (IS6, 100.00 mg/L), benzyl alcohol-d7 (IS7, 100.00 mg/L) with final concentrations. Then, 5 mL of CH_2_Cl_2_ was added, vortexed at 800 rpm for 5 min (repeated 3 times), and concentrated to 1 mL as mentioned above in the LLE method. GC-MS was performed as previously described for DB-FFAP in Section 2.4.2.

#### 2.5.2. Liquid-Liquid Extraction Combined with Gas Chromatography-Mass Spectrometry (LLE-GC-MS)

Thirty-one compounds were quantified using LLE-GC-MS. Each sample (50 mL) was diluted to 10% ethanol (*v*/*v*), saturated with NaCl, and mixed with 50 μL ISs identical to the LLME-GC-MS method. The extraction process using the LLE method is described in Section 2.4.1. The condition of GC-MS was performed as previously described on DB-FFAP in Section 2.4.2.

### 2.6. Determination of Odor Thresholds

In this study, the odor thresholds for most odorants were reported in published papers (in ethanol/water solution) to calculate the OAVs. Liu, et al. [26] confirmed that the measurements of odor thresholds determined by the ten-sample test (TST) and three-alternative forced-choice (3-AFC) method did not differ significantly or negligibly. Thus, using the TST method to determine the odor thresholds of the other odorants at 7 concentrations in 45% vol ethanol/water refers to a previously described method [27]. These compound’s odor thresholds were determined, including 2,6-dimethyl-2,4,6-octatriene, (2,2-diethoxyethyl) benzene, ethyl 3-hexenoate, 4-allylphenol, α-santalol, α-bisabolol, and β-bisabolol.

### 2.7. Aroma Recombination Tests and Omission Tests

Aroma recombination tests were carried out in a dilute alcohol solution (45% vol) and dearomatized *Zhuyeqing* (45% vol) and compared with the corresponding real *Zhuyeqing*. Aroma omission tests were conducted only in dearomatized *Zhuyeqing* and were compared with the aroma recombination model in dearomatized *Zhuyeqing*.

**Table 3:** Information on the quantitative method, internal standard (IS), quantitative ions, and quantitative parameters of chemical standard curves, odor thresholds, concentrations, and OAVs of major aroma compounds (OAV ≥ 1) in *Zhuyeqing* and base Fenjiu samples.

Compounds	* Quantitative method	* IS	Quantitative ion (*m*/*z*)	Slope	Intercept	R^2^	Odor Threshold (μg/L)	Concentrations (μg/L)		OAV	
								*Zhuyeqing*	Base Fenjiu	*Zhuyeqing*	Base Fenjiu
ethyl cinnamate	LLME	IS7	131	1.6352	−0.0068	0.9940	0.70 [28]	290.06 ± 16.88	0.00 ± 0.00	414.37	<0.01
β-damascenone	LLE	IS4	69	2.0132	0.0243	0.9935	0.10 [29]	16.65 ± 1.35	28.53 ± 1.18	166.50	285.30
ethyl octanoate	LLME	IS2	88	1.0479	−0.0156	0.9997	12.90 [30]	1113.92 ± 120.50	2398.83 ± 50.03	86.35	185.96
ethyl hexanoate	LLE	IS2	88	0.3419	1.9693	0.9981	55.33 [29]	3107.04 ± 234.61	7115.64 ± 174.68	56.15	128.60
ethyl acetate	LLME	IS2	43	0.7268	−0.0175	0.9989	32,600.00 [30]	1,696,667.43 ± 230,056.14	2,048,863.56 ± 9325.78	52.05	62.85
d-limonene	LLME	IS4	68	1.5852	−0.2081	0.9993	34.00 [31]	1302.01 ± 42.04	0.00 ± 0.00	38.29	<0.01
ethyl butanoate	LLME	IS2	71	0.6505	−0.0021	0.9995	81.50 [29]	2596.35 ± 268.14	3214.48 ± 75.96	31.86	39.44
eugenol	LLME	IS6	164	0.9889	0.4755	0.9988	470.00 [31]	13,617.04 ± 388.13	0.00 ± 0.00	28.97	<0.01
3-methylbutyl acetate	LLE	IS2	43	2.8633	0.0002	0.9959	93.93 [29]	1305.53 ± 58.25	578.79 ± 58.54	13.90	6.16
β-myrcene	LLE	IS3	93	4.6837	0.0598	0.9932	4.90 [31]	40.32 ± 3.15	4.53 ± 0.32	8.23	0.92
ethyl pentanoate	LLE	IS2	85	0.8355	−0.0044	0.9992	26.80 [30]	144.23 ± 8.13	205.44 ± 7.14	5.38	7.67
phenylacetaldehyde	LLME	IS7	91	1.3633	−0.0008	0.9982	25.00 [32]	114.59 ± 9.99	263.06 ± 4.88	4.58	10.52
2-methyl-1-propanol	LLME	IS1	43	0.4691	−0.0082	0.9998	1045.47 [29]	4666.62 ± 24.52	8787.20 ± 334.79	4.46	8.41
3-methylbutanoic acid	LLE	IS5	60	0.0669	−0.0499	0.9995	28,300.00 [30]	114,253.65 ± 5675.61	175,888.78 ± 1010.46	4.04	6.22
bornyl acetate	LLME	IS4	95	1.0059	−0.0020	0.9953	75.00 [31]	210.51 ± 5.47	0.00 ± 0.00	2.81	<0.01
guaiacol	LLME	IS6	109	1.6209	−0.0107	0.9993	9.50 [17]	23.70 ± 0.39	61.39 ± 1.38	2.50	6.46
1-nonanol	LLE	IS1	56	7.6307	−0.0557	0.9987	50.00 [33]	125.23 ± 20.3	213.25 ± 26.12	2.50	4.27
3-methyl-1-butanol	LLME	IS1	55	0.5552	0.2263	0.9993	179,000.00 [30]	396,642.77 ± 25,104.53	581,270.14 ± 22,380.84	2.22	3.25
phenol	LLME	IS7	94	2.5256	0.0045	0.9979	30.00 [17]	59.43 ± 4.05	34.23 ± 0.04	1.98	1.14
ethyl 2-hydroxybutanoate	LLME	IS2	59	0.5596	−0.0007	0.9976	800.00 [31]	1441.25 ± 82.99	730.12 ± 18.59	1.80	0.91
*trans*-isoeugenol	LLE	IS6	164	−0.0210	0.1110	0.9930	22.54 [29]	35.53 ± 1.62	0.00 ± 0.00	1.62	<0.01
ethyl 3-phenylpropanoate	LLME	IS7	104	0.1195	0.0001	0.9965	125.00 [30]	192.95 ± 37.03	600.15 ± 2.11	1.54	4.80
linalool	LLE	IS3	71	0.0294	0.4743	0.9918	30.00 [31]	29.50 ± 0.27	0.00 ± 0.00	0.98	<0.01
(-)-myrtenol	LLE	IS4	79	19.5732	−0.0910	0.9967	7.00 [31]	6.34 ± 0.85	2.63 ± 0.06	0.91	0.38
butanoic acid	LLME	IS5	60	1.2345	−0.0081	0.9996	964.00 [30]	613.63 ± 103.31	826.73 ± 23.23	0.64	0.86
β-cyclocitral	LLE	IS4	137	0.0792	0.0001	0.9943	5.00 [31]	2.65 ± 0.23	0.82 ± 0.12	0.53	0.16
(+)-2-bornanone	LLME	IS4	95	1.2116	0.0010	0.9954	1470.00 [31]	674.80 ± 29.53	0.00 ± 0.00	0.46	<0.01
acetic acid	LLME	IS5	43	0.7482	−0.5177	0.9930	160,000.00 [30]	71,164.03 ± 6150.23	70,146.27 ± 2227.35	0.44	0.44
4-ethylguaiacol	LLME	IS6	137	3.0432	−0.0171	0.9993	123.00 [32]	41.70 ± 0.91	96.50 ± 1.28	0.34	0.78
nonanal	LLE	IS2	57	2.2321	−0.0998	0.9954	122.45 [29]	40.59 ± 3.46	37.64 ± 1.01	0.33	0.31
2-phenylethyl acetate	LLME	IS7	104	2.6561	−0.0008	0.9986	200.00 [34]	58.48 ± 7.37	197.95 ± 1.80	0.29	0.99
2-furanmethanol	LLME	IS7	98	0.6940	−0.0072	0.9983	2000.00 [35]	545.01 ± 59.82	35.42 ± 12.88	0.27	0.02
hexanoic acid	LLME	IS5	60	1.4758	−0.0786	0.9963	2520.00 [32]	667.35 ± 58.42	804.02 ± 84.03	0.26	0.32
vanillin	LLE	IS6	151	6.2539	−0.0601	0.9984	438.52 [29]	106.70 ± 0.03	3.14 ± 0.30	0.24	0.01
4-vinylguaiacol	LLE	IS6	150	0.9611	−0.0080	0.9989	209.30 [29]	47.27 ± 0.19	4.96 ± 0.27	0.23	0.02
creosol	LLME	IS6	138	1.6221	−0.0048	0.9988	315.00 [32]	65.23 ± 0.74	90.44 ± 3.17	0.21	0.29
α-santalol	LLE	IS4	69	0.1410	−0.0006	0.9936	1193.25 ^a^	138.00 ± 0.12	0.00 ± 0.00	0.12	<0.01
benzaldehyde	LLME	IS7	106	0.9835	0.0003	0.9984	515.00 [36]	56.25 ± 7.01	95.94 ± 4.57	0.11	0.19
α-ionone	LLE	IS4	121	1.3452	0.0016	0.9942	13.70 [37]	1.54 ± 0.15	0.00 ± 0.00	0.11	<0.01
geraniol	LLE	IS4	69	0.0942	−0.0005	0.9931	80.00 [38]	7.35 ± 0.46	4.32 ± 0.02	0.09	0.05
nerolidol	LLE	IS3	69	1.0404	−0.0109	0.9990	250.00 [31]	21.03 ± 1.93	7.37 ± 0.79	0.08	0.03
ethyl 2-phenylacetate	LLME	IS7	91	2.5002	−0.0005	0.9983	407.00 [30]	31.97 ± 2.02	70.07 ± 1.55	0.08	0.17
(2,2-diethoxyethyl) benzene	LLME	IS7	103	1.1973	−0.0003	0.9984	995.50.00 ^a^	67.37 ± 6.18	209.08 ± 3.72	0.07	0.21
2-phenylethanol	LLME	IS7	91	2.7553	0.4170	0.9933	40,000.00 [34]	2368.18 ± 40.49	3687.14 ± 131.73	0.06	0.09
α-bisabolol	LLE	IS4	109	0.2030	−0.0026	0.9825	756.42.00 ^a^	44.50 ± 0.27	0.00 ± 0.00	0.06	<0.01
furfural	LLME	IS7	96	0.7745	0.0093	0.9967	39,000.00 [31]	1838.44 ± 10.45	4220.33 ± 171.02	0.05	0.11
*p*-cresol	LLE	IS6	107	2.2287	−0.0035	0.9995	166.97 [29]	8.31 ± 0.26	9.56 ± 0.42	0.05	0.06
β-caryophyllene	LLE	IS4	93	0.0163	0.0055	0.9876	150.00 [31]	6.20 ± 0.72	0.65 ± 0.15	0.03	<0.01
γ-terpinene	LLE	IS4	93	2.5737	0.0970	0.9939	1000.00 [31]	33.90 ± 3.23	0.00 ± 0.00	0.03	<0.01
ethyl nonanoate	LLE	IS2	88	1.2116	0.0084	0.9982	3150.00 [30]	70.87 ± 6.67	204.84 ± 6.76	0.02	0.07
ethyl 3-hexenoate	LLE	IS7	69	2.4232	−0.0094	0.9982	289.75 ^a^	8.43 ± 0.89	5.42 ± 0.47	0.03	0.02
isoamyl lactate	LLME	IS2	45	0.4669	0.0188	0.9971	131,703.40 [33]	1932.33 ± 133.66	3402.51 ± 251.35	0.01	0.03
2-nonanol	LLE	IS1	45	0.6103	0.0023	0.9998	75.00 [39]	0.68 ± 0.12	2.87 ± 0.42	0.01	0.04
4-allylphenol	LLE	IS6	134	1.6081	0.0112	0.9987	2934.70 ^a^	22.51 ± 0.82	0.00 ± 0.00	0.01	<0.01
β-bisabolol	LLE	IS4	82	0.1757	0.0019	0.9939	1948.01 ^a^	19.50 ± 1.20	0.00 ± 0.00	0.01	<0.01
β-ionone	LLME	IS4	177	2.0709	0.0001	0.9993	963.00 [40]	6.25 ± 0.47	0.00 ± 0.00	0.01	<0.01
terpinen-4-ol	LLE	IS4	71	0.0148	0.0452	0.9968	1540.00 [16]	7.82 ± 0.24	0.45 ± 0.12	<0.01	<0.01
benzyl alcohol	LLME	IS7	108	1.1002	−0.0018	0.9950	40,900.00 [28]	61.06 ± 6.62	76.97 ± 4.15	<0.01	<0.01
2,3,5,6-tetramethylpyrazine	LLME	IS7	136	2.3661	0.0023	0.9978	80,073.16 [29]	13.30 ± 2.80	60.70 ± 4.30	<0.01	<0.01
isoamyl isovalerate	LLE	IS7	70	0.0469	−0.0031	0.9908	1000.00 [31]	3.02 ± 0.21	1.59 ± 0.05	<0.01	<0.01
caryophyllene oxide	LLE	IS4	79	0.4438	0.1134	0.9950	410.00 [31]	0.62 ± 0.09	0.00 ± 0.00	<0.01	<0.01
2,6-dimethyl-2,4,6-octatriene	LLE	IS3	121	0.0616	0.0035	0.9921	2137.24 ^a^	2.05 ± 0.07	0.00 ± 0.00	<0.01	<0.01

* Quantitative method: “LLME” stands for liquid-liquid microextraction with gas chromatography-mass spectrometry; “LLE” stands for liquid-liquid extraction with gas chromatography-mass spectrometry. * IS: the internal standard used to quantitate the compounds: 1-butanol-d10 (IS1), ethyl octanoate-d15 (IS2), (±)-linalool-d3 (IS3), l-menthol (IS4), 2-ethylbutyric acid (IS5), 2-methoxyphenol-d3 (IS6), benzyl alcohol-d7 (IS7). ^a^ Odor thresholds were calculated in 45% ethanol/water detected in this study.

#### 2.7.1. Aroma Recombination Tests by Descriptive Analysis

A dilute alcohol solution was prepared using ethanol and microfiltered water to obtain an ethanol level of 45% vol as a model solution. Following the methodology outlined in [41], dearomatized *Zhuyeqing* was prepared by evaporation using a Rotavapor (RV 10 digital V Rotary Evaporators, IKA, Marseille, Germany) with a bath temperature of 20 °C to two-thirds of its original volume. The evaporated liquid was then mixed with ethanol and microfiltered water to match the volume and alcohol concentration of the original *Zhuyeqing*. The dearomatized *Zhuyeqing* was further treated with 5 g/L LiChrolut EN resin (40–120 μm) and stirred for 12 h. The headspace-gas chromatography-mass spectrometry (HS-GC-MS) test confirmed that the resulting dearomatized *Zhuyeqing* did not contain any trace of the compounds included in this study. The sensory test confirmed that dearomatized *Zhuyeqing* had a very low-intensity neutral aroma that could barely be perceived.

The aroma compounds with OAVs ≥ 1 in *Zhuyeqing* were added to the model solution, dearomatized *Zhuyeqing* based on their actual concentrations (Table 3) to create a recombination model, and then compared with the original features of real *Zhuyeqing*. The simulated aroma model and real *Zhuyeqing* samples were evaluated by a panel of 10 assessors, as mentioned in the sensory analysis (Section 2.3). Assessors scored the perception strength of the seven aroma profiles of *Zhuyeqing* from 0 (very weak) to 6 (very strong).

#### 2.7.2. Omission Tests by Discrimination Analysis

According to a previously published method [2], 22 omission (Table 4) tests were prepared to ascertain the significance of these compounds using a triangle test. Three samples (~20 mL) were simultaneously placed in glasses for evaluation, containing one omission model and two recombination models. The 10 judges were required to identify the sample with the most noticeable perceptual difference from the other two. Each test was repeated in triplicate, and the results were statistically analyzed.

### 2.8. Statistical Analysis

Statistical analyses were carried out using SPSS (Chicago, IL, USA) version 26.0, for Windows. The aroma profiling data were assessed by analysis of variance (ANOVA) with SPSS.

## 3. Results and Discussion

### 3.1. Sensory Analysis of Zhuyeqing and Base Fenjiu Samples

**Figure 2:**
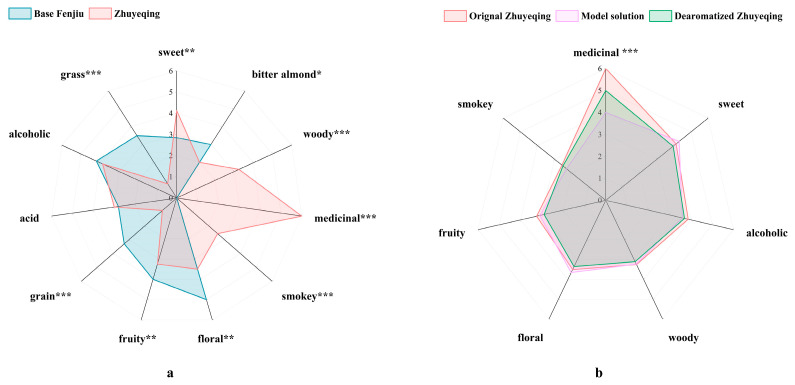
(**a**) Aroma profiles of *Zhuyeqing* and base Fenjiu samples; (**b**) Aroma profile analyses of *Zhuyeqing* and the complete aroma reconstitution model in model solution (45% vol ethanol) and dearomatized *Zhuyeqing* (45% vol ethanol). “*”, “**”, and “***” indicate significance at *p* < 0.05, 0.01, and 0.001, respectively.

Aroma profiling analyses were performed to preliminarily assess the overall difference in aroma profiles between *Zhuyeqing* and base Fenjiu, and 11 odor features that agreed with the sensory group were used. The results shown in Figure 2a reveal significant differences between the *Zhuyeqing* and base Fenjiu samples. The general aroma profiles of *Zhuyeqing* were richer than those of the base Fenjiu. Statistical analysis showed that the medicinal, woody, smokey, sweet, grass, grain, fruit, and floral aroma features were significantly different (*p* < 0.01) between the two samples. Of these, medicinal, woody, and smokey aromas were unique aroma characteristics of *Zhuyeqing* compared to the base Fenjiu, and sweet intensities were higher in *Zhuyeqing*, but grain, grass, floral, and fruit intensities were higher in base Fenjiu. To further explain the factors causing the differences in aroma between *Zhuyeqing* and the base Fenjiu, GC-O was used to study the odor differences in these two samples.

### 3.2. Identification of Aroma-Active Compounds in Zhuyeqing and Base Fenjiu Samples

The aroma extracts of *Zhuyeqing* and base Fenjiu were compared by AEDA to obtain the FD value of each aroma compound that could be detected by GC-O (Figure S1). One hundred and two odorants were confirmed by comparing their RIs on DB-FFAP and DB-5 columns, odor characteristics, and/or mass spectra with reference compounds. These substances included 27 esters, 24 terpenes, 14 alcohols, 12 phenols, 8 aldehydes, six acids, two lactones, three furans, two ketones, one pyrazine, and three other compounds (Table 2).

In *Zhuyeqing*, 93 odorants were detected, and 64 odorants had FD ≥ 32. The highest FD factors (FD ≥ 1024) were obtained for eugenol (92, clove), 4-vinylguaiacol (94, smokey), and trans-isoeugenol (96, clove). In addition, ethyl octanoate (35, pear), β-damascenone (73, honey, floral, apple-like), ethyl cinnamate (89, cinnamon), α-santalol (98, woody), vanillin (101, vanillin), acetic acid (38, vinegar), furfural (40, almond), linalool (46, floral), 3-methylbutanoic acid (59, sour), geraniol (74, rose), and β-ionone (81, orris) had higher FD factors (FD ≥ 256) (Table 2). In the base Fenjiu, a total of 73 odorants were detected, and 27 odorants had FD ≥ 32. Among these compounds, ethyl butanoate (11, pineapple), ethyl hexanoate (22, fruity), and β-damascenone (73, honey, floral, apple-like) displayed the highest FD of 512, followed by 2-phenylethyl acetate (72, floral), and ethyl 3-phenylpropanoate (78, floral). These odorants may be the main ingredients responsible for the distinctive aroma characteristics of *Zhuyeqing* and base Fenjiu. Among all odorants with FD ≥ 32, 22 odorants were detected only in *Zhuyeqing*, and most of them were terpenes and phenols, such as eugenol (FD = 2048), trans-isoeugenol (FD = 1024), α-santalol (FD = 512), linalool (FD = 256), and β-ionone (FD = 256).

Aroma profiling analyses of *Zhuyeqing* and base Fenjiu showed that medicinal, smokey, woody, and sweet aromas were developed after adding the infusion extracts of 12 traditional Chinese herbs to the base Fenjiu, creating a more complex flavor. In addition to the previously published important compounds in FDS rich in herbal extracts [15,16,17,40], this part of the study newly detected some aroma compounds that may be due to extracts with different types of herbals. These included ethyl 3-hexenoate (27, fruity), 2,6-dimethyl-2,4,6-octatriene (30, herbal), β-cyclocitral (55, sweet), endo-borneol (66, woody), geranyl acetate (69, rose), geraniol (74, rose), ethyl cinnamate (89, cinnamon), β-bisabolol (90, citrus), 4-vinylguaiacol (94, smokey), and α-bisabolol (95, floral).

### 3.3. Quantification of Aroma Compounds and OAV Analysis

Except for ethyl dl-2-hydroxycaproate and ethyl 2-hydroxy-3-methylbutanoate, which could not find standards, a total of 62 aroma compounds (FD > 32) were quantified using various quantification methods. An equal amount of internal standards for calibration was added during the extraction of the aroma compounds, and the construction of standard curves to compensate for the loss of compounds in the course of extraction and to ensure the accuracy of the quantitative results. Quantitative results combined with odor activity values (OAV, the ratio between concentration and odor threshold) of each volatile component were used to explain the causes of the olfactory differences and to verify the contributions of these aroma compounds to the aromas of the *Zhuyeqing* and base Fenjiu samples (Table 3). Quantitative analysis showed that ethyl acetate had the highest concentrations in the *Zhuyeqing* and base Fenjiu samples (>1.60 and >2.00 g/L, respectively), followed by 3-methyl-1-butanol (397 and 581 mg/L, respectively), and 2-methyl-1-propanol (114 and 176 mg/L, respectively).

Among the 62 aroma compounds quantified, only 22 were further confirmed as important aromas in *Zhuyeqing,* with OAVs ≥ 1.00. This difference may be the result of two reasons: (1) FD was obtained from the air, and the odorant thresholds in the air were lower than those in the other matrixes; and (2) the thresholds listed in Table 3 were derived either from Baijiu, Whiskey, or ethanol solution systems, and no literature has reported these odorant thresholds in the *Zhuyeqing* matrix.

Ethyl cinnamate, β-damascenone, ethyl octanoate, ethyl acetate, and ethyl hexanoate had the highest OAVs of ≥50.00. d-limonene, ethyl butanoate, eugenol, and 3-methylbutyl acetate had OAVs > 10.00. The compound with the highest OAV of 414.37 was ethyl cinnamate, followed by β-damascenone (OAV = 166.50) and ethyl octanoate (OAV = 86.35); their OAVs were much higher than those of other compounds. Ethyl cinnamate (FD = 512), β-damascenone (FD = 512), and ethyl octanoate (FD = 512) also had higher FD values in GC-O analysis. Thus, ethyl cinnamate, β-damascenone, and ethyl octanoate should be regarded as important aroma compounds in *Zhuyeqing*. Ethyl cinnamate with cinnamon, sweet, and fruity notes was confirmed as a key aroma in Huangjiu [42]. β-damascenone, which contributes to the fruity character (apple, honey, citrus, rose, pear), was demonstrated to be a key food odorant in many natural foods [43], such as grapefruit juice [44], wine [45], flavored wine [4] and Baijiu [30]. Dunkel et al. [43] concluded that ethyl octanoate is a key odorant in all alcoholic beverages. This compound is also a key aroma in *Zhuyeqing*. In addition, eugenol, trans-isoeugenol, ethyl hexanoate, d-limonene, ethyl butanoate, and 3-methylbutyl acetate had higher FDs ≥ 128, and all of their OAVs ≥ 1.00. These compounds could also be important aroma compounds in *Zhuyeqing* and might give its possible characteristics, such as sweetness, fruit, and floral. Eugenol and trans-isoeugenol are mainly derived from clove, emitting smokey, clove, and spice aromas, which are also important odorants in Chinese *JingJiu* [17] and *Wujiapi* [16]. The use of 12 Chinese herbs also brings some flavors that are different from common liquor samples and resulted in some compounds with high FD and low OAV, such as geraniol (FD = 256, OAV < 1), β-caryophyllene (FD = 62, OAV < 1), caryophyllene oxide (FD = 32, OAV < 1), 2-furanmethanol (FD = 128, OAV < 1), and nerolidol (FD = 32, OAV < 1).

Similarly, sixteen important odorants in base Fenjiu had OAVs of ≥1.00, and β-damascenone, ethyl octanoate, ethyl hexanoate, ethyl acetate, and ethyl butanoate had the highest OAVs of ≥10.00. Phenylacetaldehyde, 3-methylbutanoic acid, 2-methyl-1-propanol, guaiacol, 3-methylbutyl acetate, ethyl 3-phenylpropanoate, 1-nonanol, 3-methyl-1-butanol, ethyl decanoate, and phenol had OAVs ≥ 1.00. β-damascenone (OAV = 287.46), ethyl octanoate (OAV = 185.96), and ethyl hexanoate (OAV = 128.6) were the most important key aroma compounds with higher FD values in GC-O analysis. All sixteen aroma compounds (OAV ≥ 1.00) in the base Fenjiu had higher OAVs than those in the *Zhuyeqing* (except 3-methylbutyl acetate). Therefore, these compounds in *Zhuyeqing* may be derived from the base Fenjiu.

Only seven aroma compounds, ethyl cinnamate, d-limonene, eugenol, 3-methylbutyl acetate, β-myrcene, bornyl acetate, and ethyl 2-hydroxybutanoate, had higher OAVs (>1) in *Zhuyeqing* than in the base Fenjiu.

### 3.4. Aroma Recombination

To validate the aroma contributions of odorants determined by AEDA and OAV analysis, compounds with OAV ≥ 1 to *Zhuyeqing* were recombined in dearomatized *Zhuyeqing* (45% vol ethanol) and model solution (45% vol ethanol) and compared with the corresponding real *Zhuyeqing* by trained panelists.

As shown in Figure 2b, compared to the original *Zhuyeqing*, there were only minor differences between the recombinant model and the original sample regarding the seven aroma descriptors (except medicinal). The aroma strength of the dearomatized *Zhuyeqing* reconstitution was more similar to that of the model solution reconstitution, suggesting that the flavor profile of the dearomatized *Zhuyeqing* reconstitution was simulated more successfully than that of the model solution reconstitution. Therefore, the overall aroma perception of *Zhuyeqing* can be affected by its matrix. In addition, the intensity of the medicinal flavor was still significantly different from that of the original *Zhuyeqing*. This may be due to the composition complexity of FDS, making overlapping chromatographic peaks that lead to missing key compounds. Thus, the medicinal aroma characteristic will be further investigated in the next study.

### 3.5. Omission Tests

To investigate the potential contributions of these odorants, 22 omitted models were created (Table 4). The differences between each of the omitted models and the full model were compared by a triangle test. The results revealed that M1 (esters), M2 (terpenes), and M3 (phenols) were the key groups in *Zhuyeqing*. All the assessors could detect the difference when missing these groups, indicating their highly significant influence on the overall aroma. However, only four of the ten evaluators could correctly distinguish the omission of M4 (all acids) and M5 (all alcohols). Perhaps the presence of abundant terpenes and phenols affects their perception and makes them insignificant in the overall profile.

When M1-1 (ethyl cinnamate), M1-2 (ethyl octanoate), and M1-3 (ethyl acetate) with fruity notes were omitted, very high significance was observed in the evaluation. M1-6 (3-methylbutyl acetate) showed a significant difference. M2-1 (β-damascenone), with honey, floral, and apple-like notes, was the most important terpene with a highly significant influence on the overall aroma. M2-4 (bornyl acetate), with woody, herbal, and cool notes, was an important odor that affected the overall aroma with highly significant differences. M3-1 (eugenol), with sweet, clove, and spice notes, was the most important phenol with a highly significant influence on the overall aroma and medicinal characterization. M3-2 (guaiacol) had a highly significant influence on the medicinal aroma. However, when the remaining compounds were omitted, no significant differences were observed. This means that these compounds were not the most important aroma contributors or they affected the overall profile of the odor interaction.

*Zhuyeqing* is a very complex system. Our current research has enriched the flavor theoretical system of *Zhuyeqing*, even the Chinese traditional flavored distilled liquor. The medicinal character of *Zhuyeqing* will be a direction for our future research on *Zhuyeqing*. In addition, the aroma characteristics of *Zhuyeqing* during aging remain mysterious and need to be investigated.

## 4. Conclusions

This study provides a preliminary understanding of the aroma compounds in *Zhuyeqing* and Fenjiu. Sixty-four odorants (FD > 32) were confirmed by GC-O. Using various quantification methods and calculating the OAVs, 22 odorants had OAVs > 1. These compounds were reconstituted in the model solution (45% vol ethanol) and dearomatized *Zhuyeqing* (45% vol ethanol), and the overall aroma characteristics of *Zhuyeqing* were better reconstituted in the dearomatized *Zhuyeqing*. Recombination and omission model sensory studies confirmed that the compounds of esters, terpenes, and phenol groups have an important influence on the overall aroma of *Zhuyeqing*. Among these, ethyl cinnamate, ethyl octanoate, ethyl acetate, β-damascenone, and eugenol were confirmed to be the key aroma components of *Zhuyeqing*.

In this paper, the matrix of *Zhuyeqing* had a very important effect on its aroma, and the medicinal character intensity of *Zhuyeqing* was still significantly different from that of the original *Zhuyeqing*.

## Figures and Tables

**Table 1:** Definitions and references of aroma attributes.

No.	Aroma	Definition	Reference (in 45% *v*/*v* Ethanol/Water)
1	medicinal	aroma of herbs	ethyl 2-hydroxybutanoate (50 mg/L)
2	sweet	aroma similar to honey and sweet fruits	*β*-damascenone (100 μg/L)
3	alcoholic	aromas presented by alcohols	45% *v*/*v* ethanol/water
4	woody	like wood	extraction of Muxiang
5	floral	similar to the aroma of florals	extraction of Juhua
6	fruity	aroma like ripe fruits	ethyl acetate (2 g/L) and ethyl hexanoate (10 mg/L)
7	smokey	similar to smoke	sotolon (1 mg/L)
8	grain	aroma obtained by fermenting and distilling sorghum, rice, and wheat.	cooked sorghum
9	acid	aroma presented by volatile acidic components	acetic acid (1 g/L)
10	grass	grass-like aroma	hexanal (2 mg/L)
11	bitter almond	aroma presented by bitter almond	furfural (50 mg/L)

**Table 2:** The FD factors of the aroma compounds in *Zhuyeqing* and base Fenjiu.

No ^a^	CAS	Compounds ^b^	RI ^c^		RT	Odor Descriptor ^d^	Identification	FD Factor ^e^	
			FFAP	DB-5	DB-5			*Zhuyeqing*	Base Fenjiu
1	75-07-0	acetaldehyde	700	415	1.008	pungent, ethereal	MS, RI, Odor, Std	4	8
2	123-38-6	propanal	810	515	1.050	pungent, ethereal	MS, RI, Odor, Std	4	8
3	123-72-8	butanal	870	545	1.065	fresh, aldehydic	MS, RI, Odor, Std	16	8
4	141-78-6	ethyl acetate	870	612	1.453	pineapple, fruity	MS, RI, Odor, Std	64	32
5	105-57-7	1,1-diethoxyethane	898	730	2.117	fruity	MS, RI, Odor, Std	2	64
6	105-37-3	ethyl propanoate	965	737	2.241	fruity	MS, RI, Odor, Std	1	32
7	431-03-8	2,3-butanedione	989	nd	nd	buttery	MS, RI, Odor, Std	n.d	1
8	97-62-1	ethyl 2-methylpropanoate	998	760	2.725	floral, fruity	MS, RI, Odor, Std	8	16
9	78-92-2	2-butanol	1005	615	1.523	fruity	MS, RI, Odor, Std	n.d	32
10	71-23-8	propanol	1015	575	1.125	alcoholic, plant	MS, RI, Odor, Std	n.d	16
11	105-54-4	ethyl butanoate	1035	791	3.362	pineapple, fruity	MS, RI, Odor, Std	128	512
12	108-64-5	ethyl 3-methylbutanoate	1075	855	4.681	sweet, fruity	MS, RI, Odor, Std	n.d	16
13	78-83-1	2-methyl-1-propanol	1086	705	1.746	mellow, nail polish	MS, RI, Odor, Std	32	16
14	66-25-1	hexanal	1100	785	3.263	green	MS, RI, Odor, Std	n.d	8
15	71-36-3	butanol	1110	670	1.654	alcoholic	MS, RI, Odor, Std	n.d	1
16	123-92-2	3-methylbutyl acetate	1122	867	4.956	banana	MS, RI, Odor, Std	128	64
17	539-82-2	ethyl pentanoate	1134	894	5.508	fruity, strawberry	MS, RI, Odor, Std	128	32
18	123-35-3	β-myrcene	1161	987	7.930	grassy, woody	MS, RI, Odor, Std	128	n.d
19	137-32-6	2-methyl-1-butanol	1190	743	2.363	alcoholic	MS, RI, Odor, Std	8	16
20	123-51-3	3-methyl-1-butanol	1198	748	2.465	banana, ether	MS, RI, Odor, Std	64	16
21	5989-27-5	d-limonene	1203	1024	8.946	orange, lemon	MS, RI, Odor, Std	128	n.d
22	123-66-0	ethyl hexanoate	1234	995	8.197	sweet, fruity, pineapple	MS, RI, Odor, Std	128	512
23	71-41-0	pentanol	1240	766	2.851	fruity, alcoholic	MS, RI, Odor, Std	n.d	2
24	99-85-4	γ-terpinene	1244	1059	9.952	gasoline, turpentine	MS, RI, Odor, Std	128	n.d
25	659-70-1	isoamyl isovalerate	1294	nd	nd	sweet, fruity, green, apple	MS, RI, Odor, Std	32	2
26	7789-92-6	1,1,3-triethoxypropane	1298	1075	10.401	fruity	MS, RI, Odor, Std	8	4
27	2396-83-0	ethyl 3-hexenoate	1303	nd	nd	fruity, pineapple, green, candy	MS, RI, Odor, Std	64	1
28	513-86-0	acetoin	1349	731	2.149	creamy, buttery	MS, RI, Odor, Std	16	2
29	111-27-3	hexanol	1366	859	4.763	ethereal, fusel	MS, RI, Odor, Std	2	4
30	673-84-7	2,6-dimethyl-2,4,6-octatriene	1372	nd	nd	spices, nutty, skin, peppery, herbal	MS, RI, Odor, Std	32	n.d
31	97-64-3	ethyl lactate	1382	815	3.862	fatty, pineapple	MS, RI, Odor, Std	8	4
32	124-19-6	nonanal	1393	1101	11.133	citrus-like, soapy	MS, RI, Odor, Std	128	32
33	3391-86-4	1-octene-3-ol	1410	980	7.745	soapy, mushroom	MS, RI, Odor, Std	n.d	1
34	52089-54-0	ethyl 2-hydroxybutanoate	1427	900	5.621	floral, fruity	MS, RI, Odor, Std	128	64
35	106-32-1	ethyl octanoate	1434	1194	13.756	pear, lychee	MS, RI, Odor, Std	512	256
36	2441-06-7	ethyl 2-hydroxy-3-methylbutanoate	1441	959	7.197	pineapple-like	MS, RI, Odor, Std	64	32
37	616-09-1	propyl lactate	1451	nd	nd	winey, yogurt, milky	MS, RI, Odor, Std	1	4
38	64-19-7	acetic acid	1459	696	1.703	vinegar	MS, RI, Odor, Std	256	64
39	585-24-0	isobutyl lactate	1471	978	7.693	buttery, fruity, caramellic	MS, RI, Odor, Std	8	8
40	98-01-1	furfural	1473	822	3.998	almond	MS, RI, Odor, Std	256	8
41	1124-11-4	2,3,5,6-tetramethylpyrazine	1492	1085	10.676	roasted, nutty	MS, RI, Odor, Std	32	n.d
42	628-99-9	2-nonanol	1514	nd	nd	cucumber	MS, RI, Odor, Std	32	64
43	464-49-3	(+)-2-bornanone	1519	1138	12.202	camphoraceous	MS, RI, Odor, Std	32	n.d
44	100-52-7	benzaldehyde	1532	951	6.967	almond	MS, RI, Odor, Std	32	8
45	123-29-5	ethyl nonanoate	1534	1294	16.394	fruity, grassy, grape	MS, RI, Odor, Std	64	32
46	78-70-6	linalool	1542	1099	11.078	floral, woody	MS, RI, Odor, Std	256	n.d
47	6946-90-3	ethyl dl-2-hydroxycaproate	1545	1055	9.848	floral, herbal	MS, RI, Odor, Std	128	32
48	79-09-4	propionic acid	1548	700	1.719	fruity, creamy	MS, RI, Odor, Std	16	8
49	76-49-3	bornyl acetate	1569	1282	16.043	woody, herbal	MS, RI, Odor, Std	32	n.d
50	19329-89-6	isoamyl lactate	1571	1064	10.091	fruity, creamy, nutty	MS, RI, Odor, Std	128	64
51	513-85-9	2, 3-butanediol	1579	796	3.463	fruity, creamy	MS, RI, Odor, Std	n.d	1
52	620-02-0	5-methylfurfural	1585	972	7.532	sweet, caramel	MS, RI, Odor, Std	16	2
53	87-44-5	β-caryophyllene	1598	1418	19.608	woody, spices, citrus	MS, RI, Odor, Std	64	n.d
54	562-74-3	terpinen-4-ol	1600	1173	13.181	woody, earthy, peppery	MS, RI, Odor, Std	128	n.d
55	432-25-7	β-cyclocitral	1624	nd	nd	herbal, clean, sweet, damascone	MS, RI, Odor, Std	32	n.d
56	107-92-6	butanoic acid	1631	894	5.491	cheesy, creamy	MS, RI, Odor, Std	128	32
57	110-38-3	ethyl decanoate	1638	1392	19.036	grape	MS, RI, Odor, Std	8	16
58	122-78-1	phenylacetaldehyde	1651	1040	9.402	honey-like, floral, rose	MS, RI, Odor, Std	128	16
59	503-74-2	3-methylbutanoic acid	1652	875	5.097	sour, cheese, sweaty, fruity	MS, RI, Odor, Std	256	2
60	96-48-0	butyrolactone	1653	nd	nd	milk, creamy	MS, RI, Odor, Std	8	8
61	143-08-8	1-nonanol	1656	1172	13.154	citrus, rose, fatty	MS, RI, Odor, Std	32	64
62	98-00-0	2-furanmethanol	1668	860	4.784	bitter	MS, RI, Odor, Std	128	32
63	93-89-0	ethyl benzoate	1673	1225	14.609	fruity, sweet, herbal	MS, RI, Odor, Std	8	16
64	123-25-1	ethyl succinate	1678	1171	13.107	fruity	MS, RI, Odor, Std	2	4
65	10482-56-1	(-)-α-terpineol	1696	1189	13.622	grapefruit	MS, RI, Odor, Std	8	n.d.
66	507-70-0	*endo*-borneol	1701	1160	12.818	woody, camphor, balsamic	MS, RI, Odor, Std	16	n.d.
67	6314-97-2	(2,2-diethoxyethyl) benzene	1709	1321	17.093	fresh, green, almond, sweet	MS, RI, Odor, Std	32	8
68	109-52-4	n-pentanoic acid	1736	979	7.706	cheesy, dairy-like	MS, RI, Odor, Std	8	4
69	105-87-3	geranyl acetate	1753	nd	nd	rose	MS, RI, Odor, Std	16	n.d
70	19894-97-4	(-)-myrtenol	1788	nd	nd	woody, sweet, mint, medicinal	MS, RI, Odor, Std	64	n.d
71	101-97-3	ethyl 2-phenylacetate	1790	1240	14.949	fruity, floral, cocoa	MS, RI, Odor, Std	128	64
72	103-45-7	2-phenylethyl acetate	1817	1252	15.343	fruity, floral	MS, RI, Odor, Std	128	128
73	23726-93-4	β-damascenone	1820	1382	18.609	honey, floral, apple-like	MS, RI, Odor, Std	512	512
74	106-24-1	geraniol	1842	nd	nd	rose, geranium	MS, RI, Odor, Std	256	n.d
75	142-62-1	hexanoic acid	1844	981	7.762	rotten cheesy	MS, RI, Odor, Std	32	32
76	127-41-3	α-ionone	1855	1424	19.748	orris, fruity, sweet, floral, woody	MS, RI, Odor, Std	64	n.d
77	90-05-1	guaiacol	1869	1086	10.732	spices, smokey	MS, RI, Odor, Std	128	16
78	2021-28-5	ethyl 3-phenylpropanoate	1881	1344	17.677	dried, floral	MS, RI, Odor, Std	64	128
79	100-51-6	benzyl alcohol	1884	1034	9.254	floral, phenolic	MS, RI, Odor, Std	32	16
80	60-12-8	2-phenylethanol	1919	1106	11.275	floral, rose, fresh-bread	MS, RI, Odor, Std	128	16
81	79-77-6	β-ionone	1953	nd	nd	orris, fruity, sweet, floral, woody	MS, RI, Odor, Std	256	n.d
82	93-51-6	creosol	1978	1190	13.661	smokey, spices, herbal	MS, RI, Odor, Std	128	16
83	1139-30-6	caryophyllene oxide	1992	1927	30.961	sweet, fresh, dry, woody	MS, RI, Odor, Std	32	n.d
84	108-95-2	phenol	2021	990	8.008	phenol, smokey	MS, RI, Odor, Std	64	16
85	7212-44-4	nerolidol	2036	nd	nd	floral, green, citrus, woody	MS, RI, Odor, Std	32	n.d
86	2785-89-9	4-ethylguaiacol	2037	1274	15.927	smokey, spices, herbal, woody	MS, RI, Odor, Std	128	2
87	2305-25-1	ethyl 3-hydroxyhexanoate	2046	nd	nd	fresh	MS, RI, Odor	8	4
88	106-44-5	*p*-cresol	2089	1076	10.446	phenol, smokey-like	MS, RI, Odor, Std	32	16
89	103-36-6	ethyl cinnamate	2140	1460	20.570	cinnamon, sweet and fruity notes	MS, RI, Odor, Std	512	32
90	15352-77-9	β-bisabolol	2152	1666	25.907	citrus, floral, sweet, herbal	MS, RI, Odor, Std	128	n.d
91	123-07-9	4-ethylphenol	2170	1175	13.256	spices, clove	MS, RI, Odor, Std	16	8
92	97-53-0	eugenol	2179	1353	17.970	smokey, clove, spices	MS, RI, Odor, Std	2048	n.d
93	134-96-3	syringaldehyde	2196	1651	25.480	sweet, clove	MS, RI, Odor, Std	2	n.d
94	7786-61-0	4-vinylguaiacol	2211	1308	16.838	smokey	MS, RI, Odor, Std	2048	32
95	515-69-5	α-bisabolol	2221	1683	26.425	floral, peppery, balsamic, clean	MS, RI, Odor, Std	128	n.d
96	5932-68-3	*trans*-isoeugenol	2271	1445	20.243	sweet, clove, spices	MS, RI, Odor, Std	1024	n.d
97	501-92-8	4-allylphenol	2349	nd	nd	anise-like, clove	MS, RI, Odor, Std	64	32
98	115-71-9	α-santalol	2442	1674	26.131	woody, sweety, nut	MS, RI, Odor, Std	512	n.d
99	644-30-4	α-curcumene	2502	1480	21.108	herbal	MS, RI, Odor	8	n.d
100	67-47-0	5-hydroxymethylfurfural	2526	1223	14.556	smokey-like	MS, RI, Odor	16	n.d
101	121-33-5	vanillin	2601	1391	18.940	vanilla, sweet, creamy	MS, RI, Odor, Std	512	8
102	81944-08-3	*(E)*-ligustilide	2622	1730	27.655	sweet	MS, RI, Odor, Std	16	n.d

^a^ Odorants were numbered consecutively according to their retention indices on a capillary DB-FFAP column. ^b^ Odorants were identified by comparison of their odor quality, retention indices (RIs) on DB-FFAP and DB-5 capillary columns, as well as mass spectrometry (MS) data with the data of authentic standard compounds (Std). ^c^ Retention indices were determined using a homologous series of n-alkanes. ^d^ Odor quality was detected at a sniffing port. ^e^ FD factors were determined by AEDA on a capillary DB-FFAP column. n.d: not detected at the sniffing port.

**Table 4:** Omission tests from complete aroma reconstitution in the dearomatized *Zhuyeqing* model.

No.	Omitted Compounds	n/10	Significance ^a^
M1	all esters	10/10	***
M1-1	ethyl cinnamate	9/10	***
M1-2	ethyl octanoate	9/10	***
M1-3	ethyl acetate	9/10	***
M1-4	ethyl hexanoate	4/10	ns
M1-5	ethyl butanoate	3/10	ns
M1-6	3-methylbutyl acetate	7/10	*
M1-7	ethyl pentanoate	2/10	ns
M1-8	ethyl 2-hydroxybutanoate	3/10	ns
M1-9	ethyl 3-phenylpropanoate	2/10	ns
M2	all terpenes	10/10	***
M2-1	β-damascenone	10/10	***
M2-2	d-limonene	5/10	ns
M2-3	β-myrcene	3/10	ns
M2-4	bornyl acetate	8/10	**
M3	all phenols	10/10	***
M3-1	eugenol	10/10	***
M3-2	guaiacol	8/10	**
M3-3	phenol	5/10	ns
M4	all acids	4/10	ns
M4-1	3-methylbutanoic acid	5/10	ns
M5	all alcohols	4/10	ns

^a^ “*”, “**”, and “***” indicate significance at *p* < 0.05, 0.01, and 0.001, respectively, “ns” indicate no significance.

## Data Availability

The original contributions presented in this study are included in the article/Supplementary Materials. Further inquiries can be directed to the corresponding author.

## Supplementary Materials

The following supporting information can be downloaded at https://www.mdpi.com/article/10.3390/foods14030344/s1, Figure S1: FD chromatograms obtained by AEDA of the extracts of *Zhuyeqing* and base Fenjiu samples, all odorants are displayed, and numbering corresponds to that in Table 1; Table S1: information of analytical standards.
