# Supplementary material for: Characterization of Key Aroma Compounds of Zhuyeqing by Aroma Extract Dilution Analysis, Quantitative Measurements, Aroma Recombination, and Omission Studies
Source: Foods. 2025 Jan 21;14(3):344. doi: 10.3390/foods14030344 (PMC11817035; doi:10.3390/foods14030344)
Supplement: Supplementary file 1 [file foods-14-00344-s001.zip › foods-3391468-supplementary.pdf]

Supporting Information

Characterization of Key Aroma Compounds of *Zhuyeqing* by Aroma Extract Dilution Analysis, Quantitative Measurements, Aroma Recombination, and Omission Studies

Lihua Wang [1,2], Ying Han [2], Xing Zhang [2], Xiaojuan Gao [2], Yan Xu [1], Qun Wu [1,*] and Ke Tang [1,*]

[1] Laboratory of Brewing Microbiology and Applied Enzymology, State Key Laboratory of Food Science & Technology, Key Laboratory of Industrial Biotechnology of Ministry of Education, School of Biotechnology, Jiangnan University, 1800 Lihu Ave, Wuxi 214122, China; wanglh0419@126.com (L.W.); yxu@jiangnan.edu.cn (Y.X.)

[2] Laboratory of Analytical, Quality Inspection Center, Key Laboratory of Plant Extraction and Health of Chinese Lujiu (Shanxi), Shanxi Xinghuacun Fenjiu Distillery Co., Ltd., Fenyang 032205, China; fjpjbyh@163.com (X.G.)

* Correspondence: wuq@jiangnan.edu.cn (Q.W.); tandy81@jiangnan.edu.cn (K.T.); Tel.: +86-510-85918197 (K.T.); Fax: +86-510-85918201 (K.T.)

Table S1. Information of analytical standards

No.	CAS	Name	Purity	Purchased from
1	64-19-7	acetic acid	≥98%	[1] Innochem
2	79-09-4	propionic acid	≥99%	[2] Macklin
3	107-92-6	butanoic acid	≥99%	[3] Aladdin
4	109-52-4	n-pentanoic acid	≥98%	[2] Macklin
5	142-62-1	hexanoic acid	≥99%	[1] Innochem
6	503-74-2	3-methylbutanoic acid	≥97%	[1] Innochem
7	6314-97-2	(2,2-diethoxyethyl) benzene	≥98%	[2] Macklin
8	1124-11-4	2,3,5,6-tetramethylpyrazine	≥97%	[4] Sigma-Aldrich
9	141-78-6	ethyl acetate	≥99%	[1] Innochem
10	105-54-4	ethyl butanoate	≥98%	[3] Aladdin
11	110-38-3	ethyl decanoate	≥98%	[1] Innochem
12	123-66-0	ethyl hexanoate	≥98%	[1] Innochem
13	123-29-5	ethyl nonanoate	≥98%	[1] Innochem
14	103-36-6	ethyl cinnamate	≥99%	[1] Innochem
15	97-64-3	ethyl lactate	≥98%	[1] Innochem
16	19329-89-6	isoamyl lactate	≥98%	[1] Innochem
17	539-82-2	ethyl pentanoate	≥99%	[1] Innochem
18	105-37-3	ethyl propanoate	≥98%	[5] Dikma
19	106-32-1	ethyl octanoate	≥98%	[1] Innochem
20	123-92-2	3-methylbutyl acetate	≥99%	[1] Innochem
21	52089-54-0	ethyl 2-hydroxybutanoate	≥99%	[5] Dikma
22	2396-83-0	ethyl 3-hexenoate	≥99%	[4] Sigma-Aldrich
23	97-62-1	ethyl 2-methylpropanoate	≥99%	[5] Dikma
24	108-64-5	ethyl 3-methylbutanoate	≥98%	[5] Dikma
25	96-48-0	butyrolactone	≥99%	[5] Dikma
26	585-24-0	Isobutyl lactate	≥99%	[5] Dikma
27	616-09-1	propyl lactate	≥99%	[5] Dikma
28	123-25-1	ethyl succinate	≥98%	[3] Aladdin
29	2021-28-5	ethyl 3-phenylpropanoate	≥97%	[4] Sigma-Aldrich
30	101-97-3	ethyl 2-phenylacetate	≥99%	[3] Aladdin
31	2441-06-7	ethyl 2-hydroxy-3-methylbutanoate	≥99%	[2] Macklin
32	93-89-0	ethyl benzoate	≥98%	[2] Macklin
33	6946-90-3	ethyl dl-2-hydroxycaproate	≥99%	[2] Macklin
34	103-45-7	2-phenylethyl acetate	≥99%	[1] Innochem
35	659-70-1	Isoamyl isovalerate	≥99%	[1] Innochem
36	71-36-3	butanol	≥97%	[5] Dikma
37	78-92-2	2-butanol	≥99%	[4] Sigma-Aldrich
38	628-99-9	2-nonanol	≥99%	[5] Dikma
39	78-83-1	2-methylpropanol	≥99%	[5] Dikma

No.	CAS	Name	Purity	Purchased from
40	513-85-9	2, 3-butanediol	≥98%	[4] Sigma-Aldrich
41	137-32-6	2-methyl-1-butanol	≥99%	[5] Dikma
42	123-51-3	3-methyl-1-butanol	≥98%	[4] Sigma-Aldrich
43	3391-86-4	1-octene-3-ol	≥98%	[2] Macklin
44	71-41-0	pentanol	≥99%	[2] Macklin
45	71-23-8	propanol	≥99%	[2] Macklin
46	111-27-3	hexanol	≥97%	[2] Macklin
47	143-08-8	1-nonanol	≥99%	[2] Macklin
48	100-51-6	benzyl alcohol	≥99%	[2] Macklin
49	60-12-8	2-phenylethanol	≥99%	[3] Aladdin
50	98-00-0	2-furanmethanol	≥99%	[1] Innochem
51	75-07-0	acetaldehyde	≥99%	[5] Dikma
52	123-72-8	butanal	≥97%	[5] Dikma
53	66-25-1	hexanal	≥99%	[5] Dikma
54	590-86-3	3-methylbutanal	≥98%	[5] Dikma
55	123-38-6	propanal	≥98%	[2] Macklin
56	124-19-6	nonanal	≥99%	[1] Innochem
57	100-52-7	benzaldehyde	≥98%	[2] Macklin
58	122-78-1	phenylacetaldehyde	≥99%	[3] Aladdin
59	98-01-1	furfural	≥99%	[1] Innochem
60	620-02-0	5-methylfurfural	≥98%	[5] Dikma
61	513-86-0	acetoin	≥99%	[5] Dikma
62	431-03-8	2,3-butanedione	≥99%	[4] Sigma-Aldrich
63	28664-35-9	sotolon	≥99%	[2] Macklin
64	7789-92-6	1,1,3-triethoxypropane	≥99%	[5] Dikma
65	105-57-7	1,1-diethoxyethane	≥98%	[2] Macklin
66	673-84-7	2,6-dimethyl-2,4,6-octatriene	≥99%	[5] Dikma
67	562-74-3	terpinen-4-ol	≥99%	[4] Sigma-Aldrich
68	5989-27-5	d-limonene	≥99%	[5] Dikma
69	127-41-3	α-ionone	≥99%	[2] Macklin
70	23726-93-4	β-damascenone	≥99%	[2] Macklin
71	432-25-7	β-cyclocitral	≥99%	[2] Macklin
72	79-77-6	β-ionone	≥99%	[2] Macklin
73	108-95-2	phenol	≥98%	[2] Macklin
74	7212-44-4	nerolidol	≥99%	[3] Aladdin
75	87-44-5	β-caryophyllene	≥98%	[3] Aladdin
76	507-70-0	endo-borneol	≥99%	[1] Innochem
77	19894-97-4	(-)-myrtenol	≥99%	[1] Innochem
78	99-85-4	γ-terpinene	≥99%	[1] Innochem
79	106-24-1	geraniol	≥99%	[1] Innochem
80	115-71-9	α-santalol	≥98%	[2] Macklin

No.	CAS	Name	Purity	Purchased from
81	78-70-6	linalool	≥98%	[2] Macklin
82	515-69-5	α-bisabolol	≥98%	[2] Macklin
83	15352-77-9	β-bisabolol	≥98%	[2] Macklin
84	10482-56-1	(-)-α-terpineol	≥97%	[5] Dikma
85	1139-30-6	caryophyllene oxide	≥99%	[1] Innochem
86	105-87-3	geranyl acetate	≥99%	Aladdin
87	76-49-3	bornyl acetate	≥98%	[1] Innochem
88	123-35-3	β-myrcene	≥99%	[1] Innochem
89	464-49-3	(+)-2-bornanone	≥97%	[1] Innochem
90	90-05-1	guaiacol	≥97%	[1] Innochem
91	123-07-9	4-ethylphenol	≥99%	[4] Sigma-Aldrich
92	106-44-5	p-cresol	≥98%	[4] Sigma-Aldrich
93	93-51-6	creosol	≥99%	[4] Sigma-Aldrich
94	501-92-8	4-allylphenol	≥97%	[4] Sigma-Aldrich
95	2785-89-9	4-ethylguaiacol	≥99%	[4] Sigma-Aldrich
96	7786-61-0	4-vinylguaiacol	≥97%	[5] Dikma
97	97-53-0	eugenol	≥98%	[3] Aladdin
98	121-33-5	vanillin	≥99%	[1] Innochem
99	5932-68-3	trans-isoeugenol	≥99%	[2] Macklin
IS1	34193-38-9	1-butanol-d10	≥99%	[3] Aladdin
IS2	1219798-38-5	ethyl octanoate-d15	≥99%	[6] Trc
IS3	1216673-02-7	(±)-linalool-d3	≥99%	[6] Trc
IS4	2216-51-5	L-menthol	≥99%	[3] Aladdin
IS5	88-09-5	2-ethylbutyric acid	≥99%	[3] Aladdin
IS6	74495-69-5	2-methoxyphenol-d3	≥99%	[7] Tmrm
IS7	71258-23-6	benzyl alcohol-d7	≥99%	[7] Tmrm
IS8	156420-66-5	sotolon-^{13}C2	100μg/mL	[8] Zzbio

[1] Innochem: Innochem Science & Technology Co., Ltd. (Beijing, China).

[2] Macklin: Macklin Biochemical Technology Co., Ltd (Shanghai, China).

[3] Aladdin: Aladdin Biochemical Technology Co., Ltd. (Beijing, China).

[4] Sigma-Aldrich: Sigma-Aldrich Co., Ltd. (Shanghai, China).

[5] Dikma: Dikma Technologies Inc. (Beijing, China).

[6] Trc: Toronto Research Chemicals Inc. (Toronto, Canada).

[7] Tmrm: TMRM Quality Inspection Technology Co., Ltd. (Changzhou, China).

[8] Zzbio: ZZBIO CO., LTD. (Shanghai, China)

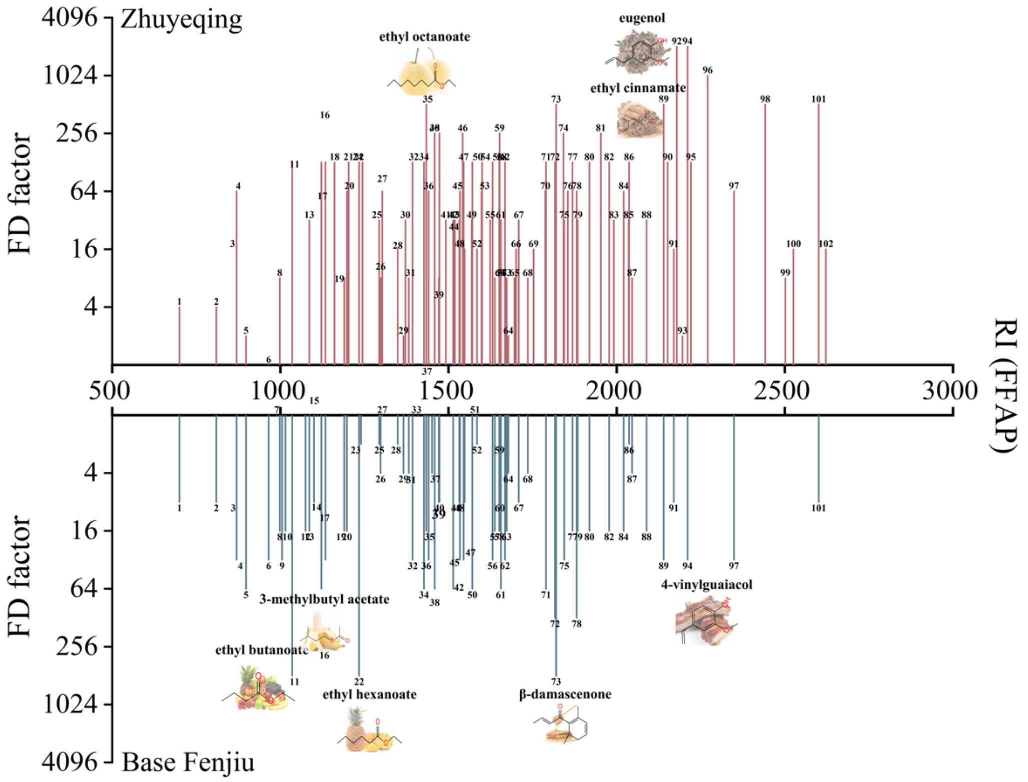

Figure S1. FD chromatograms obtained by AEDA of the extracts of *Zhuyeqing* and base Fenjiu samples. All odorants are displayed and numbering corresponds to that in Table 1.
